# A Review on Mammary Tumors in Rabbits: Translation of Pathology into Medical Care

**DOI:** 10.3390/ani9100762

**Published:** 2019-10-02

**Authors:** Sandra Schöniger, Sophie Degner, Bharat Jasani, Heinz-Adolf Schoon

**Affiliations:** 1 Targos Molecular Pathology GmbH, Germaniastrasse 7, 34119 Kassel, Germany; bharat.jasani@targos-gmbh.de; 2 Institute of Veterinary Pathology, Faculty of Veterinary Medicine, Leipzig University, 04103 Leipzig, Germany; soraehse@web.de (S.D.); schoon@vetmed.uni-leipzig.de (H.-A.S.)

**Keywords:** comparative pathology, histopathology, mammary tumors, mammary tumor-like lesions, molecular features, *Oryctolagus cuniculus*, rabbit, review

## Abstract

**Simple Summary:**

In recent years mammary cancer has been increasingly recognized in pet rabbits. In addition to uterine carcinomas—the most common tumor of female rabbits—mammary cancer can also markedly reduce the life expectancy of pet rabbits. The aim of this review is to raise awareness for these tumors and to report recent progress in related research. Their detailed characterization will likely improve medical care for affected rabbits. Moreover, study results will contribute to comparative pathology and may reveal if the rabbit is a suitable model for certain types of breast cancer in humans. Available information suggests that most invasive cancer cases develop through stepwise progression from non-invasive forms. Thus, early recognition will likely improve a complete cancer cure. So far, the only treatment option is surgical excision and prognostic factors are unknown. Recent investigations have identified tumor features with likely prognostic value. They have also revealed differences and similarities to mammary tumors in other species and breast cancer in women. Despite these initial data, continued research is necessary to gain more insights into the development of these tumors and their molecular features.

**Abstract:**

The aim of this review is to raise awareness for mammary tumors in rabbits and to report progress in related research. Currently, a standardized tumor classification for rabbits is not available, prognostic factors are unknown and the only treatment option is surgical excision. Studies showed that affected rabbits have a wide age range and are nearly exclusively female or female spayed. Most mammary tumors are carcinomas. These may occur together with non-neoplastic or benign mammary lesions. Frequent microscopic findings are lipid droplets in tumor cells, secretory activity and microscopic heterogeneity. Since carcinomas are often negative for estrogen and progesterone receptors (ER-α/PR), modulation of receptor function will unlikely be beneficial for most rabbits. ER-α and PR status may have prognostic significance, since ER-α- or PR-negative tumors have significantly higher mitotic rates than ER-α- or PR-positive tumors. The frequent secretory activity of rabbit mammary tumors may suggest an influence of prolactin on tumorigenesis. Available data contribute to comparative pathology and are the basis for future molecular studies into the identification of additional prognostic factors and novel therapeutic options. They will also reveal the suitability of the rabbit as a model for certain types of breast cancer in women.

## 1. Introduction

The aim of this review is to raise awareness for mammary tumors in rabbits and to report progress in related research.

Rabbits belong to the family of Leporidae in the order Lagomorpha [1,2]. The genetic origin of domestic rabbits is *Oryctolagus cuniculus* ssp. *cuniculus* [2,3], which has its geographic origin in the Iberian Peninsula and colonized France about 1800 years later [3,4]. Today, *O. cuniculus* shows a worldwide distribution [3,4]. Genetic studies indicate a single origin of domestication that likely occurred in French monasteries within the last 1500 years [3,4]. Most rabbit breeds, however, were established within the last 200 years in Western Europe [3,4]. Rabbit domestication was associated with a greater loss of genetic diversity as those observed in most other domesticated species [3,4]. Likely causative factors are a single origin of domestication with a small population size and only rare backcrosses with wild rabbits [4].

More than 200 different breeds of rabbit exist that show marked differences in size and body weight, ranging from approx. 1.5 to 9 kg [1,2,4]. This marked phenotypic diversity is reflected by well-defined breed-related genetic substructures [4].

Rabbits display some species-specific features [1,2]. Those related to reproduction are summarized below. They also show several traits, some of which are breed- or line-specific, making them suitable models for different aspects of biomedical research [2,3]. Rabbits are models for several non-infectious and infectious human diseases [2,3,4,5,6]. These include atherosclerosis, hypertension, cataracts and Alzheimer’s disease [2,3,4,5,6], as well as syphilis, tuberculosis and viral-induced acute hepatic failure [5]. Transgenic rabbits are available to examine human cardiovascular disease and lipoprotein metabolism [6], as well as immune responses induced by papillomavirus, human immunodeficiency virus and ocular infection with herpesvirus [5].

The most common tumor in the female rabbit is the uterine carcinoma [2,7,8]. In the past, most domestic rabbits constituted laboratory and meat rabbits, and spontaneous mammary tumors were considered as rare [9]. Today, however, these tumors seem to be diagnosed quite frequently in pet animals [10,11]. Baum and Hewicker-Trautwein [10] reported that mammary tumors constituted about 20% of the samples of pet rabbits submitted for microscopic evaluation to a diagnostic laboratory in Germany. The obvious increased occurrence of mammary tumors in rabbits is likely attributed to the increased popularity of rabbits as pets, together with their relative high life expectancy [12] that is almost comparable to that of dogs [13,14]. For the following review, PubMed and Google Search were used in addition to textbooks to obtain the data from the literature related to neoplastic and non-neoplastic mammary gland lesions in rabbits, as well as selected aspects of comparative pathology.

## 2. Reproduction in Rabbits—A Brief Summary

Since the doe is an induced ovulator, and does do not show a regular estrous cycle, mating can be arranged over the entire year [1,2,8,15]. Receptive periods of 7–14 days duration alternate with quiet phases of 1–2 days [8,15]. In addition to mating, ovulation can also be provoked by other external influences [2]. Sterile mating often leads to pseudopregnancy with the development of secretory activity of mammary tissue [1,16]. The duration of pseudopregnancy lasts about 15–18 days, whereas pregnancy has a duration of 29–35 days [1,8].

The uterus of the doe is composed of two uteri and two cervices that connect to a common vagina [1,2].

## 3. Mammary Gland–Histology and Specific Features in Rabbits

The tubuloalveolar structures of the mammary gland are lined by two types of epithelial cells, i.e., an inner layer of luminal secretory epithelial cells and an outer layer of myoepithelial cells [17,18,19].

Does contain two mammary gland chains each with 4–5 mammary glands [1,8]. The mammary gland ducts do not fuse, but open independently into the mamillae [1].

In comparison to most other pet animals, the milk of the doe has a low lactose content, whereas proteins and lipids are high [20]. During lactation, the fat content varies between 9% and 17.5%, whereas the fat content of human and cow milk is 4.6% and 4%, respectively [1].

## 4. Mammary Tumors in Rabbits

### 4.1. A Brief History

Greene [9,21,22] has to be considered the pioneer of scientific studies on rabbit mammary tumors. To the best of our knowledge, he was the first scientist that published a comprehensive investigation on mammary tumors in a breeding colony of laboratory rabbits [9,21,22]. Since these tumors occurred nearly exclusively in two different rabbit families, the involvement of hereditary factors was suspected [9,21,22]. The genetics of tumor-bearing rabbits and the mode of inheritance, however, were not examined [9,21,22].

Greene [21] described two ways of development of malignant mammary tumors. In the more prevalent form, carcinomas arise in cystic mammary glands by stepwise progression from simple cysts over intra-cystic benign papillary tumors and non-invasive cancer to invasive carcinomas with possible metastases. The time duration from the diagnosis of simple cysts to the presence of invasive carcinomas is variable ranging from two months to more than one year [21]. In the other form, carcinomas develop in mammary tissue without pre-existing cystic lesions [21]. Cystic disease as well as benign and malignant neoplasia show secretory activity [21]. Does with mammary gland lesions often display the concurrent presence of endometrial hyperplasia. A uterine carcinoma was diagnosed in four does, three with and one without preexisting cystic mammary disease [22].

### 4.2. Reported Data and Open Questions

#### 4.2.1. Anamnestic Data and Histopathological Features

Rabbits with mammary tumors show a wide age distribution, i.e., reported ages vary between 8 months and 14 years with mean ages between 4.9 to 5.5 years [9,10,11,23,24,25,26].

All rabbits with confirmed mammary gland tumors and known gender are female or female spayed [10,11,26]. In comparison, in guinea pigs, mammary tumors occur with equal frequency in female and male animals [27].

Tumors have no predilection site within the mammary tissue [10]. In bitches, however, mammary tumors are more frequently located in posterior mammary complexes [28].

**Figure 1:**
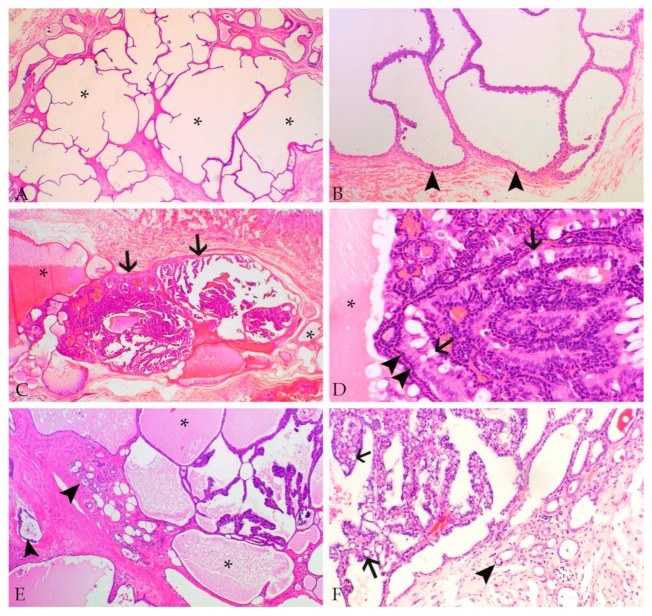
Microscopic images from rabbit mammary gland lesions (hematoxylin–eosin stain). (**A**) Multiple non-neoplastic mammary cysts (asterisks). (**B**) Cystadenoma surrounded by a thin fibrous connective tissue capsule (arrowheads). (**C**) Mammary gland with the concurrent presence of cysts containing proteinaceous secretion (asterisks) and an intraductal papillary carcinoma (arrows). (**D**) Tubulopapillary structures of the in situ carcinoma are lined by a continuous outer layer of ME cells (arrowheads) and an inner layer of neoplastic epithelial cells, some of these containing lipid droplets (arrows). The secretory material in the cystic space is indicated by an asterisk. (**E**) Tubulopapillary and cystic adenocarcinoma with secretory activity (asterisks) and moderate invasive growth in the stroma (arrowheads). (**F**) Tubulopapillary adenocarcinoma with numerous lipid droplets in tumor cells (arrows) and moderate invasive growth in the stroma (arrowhead).

Reported types of non-neoplastic proliferative mammary gland lesions, as well as benign and malignant tumors are summarized in Table 1, with some also displayed in Figure 1.

Baum and Hewicker-Trautwein [10] classify mammary tumors in pet rabbits according to the World Health Organization’s histological classification for mammary tumors in cats and dogs [29] under consideration of the modification for canine mammary tumors [30]. Considering these guidelines [29,30], adenocarcinomas are further subclassified in different histotypes according to their predominant growth pattern [10,11]. The tubular histotype is most commonly observed [10,11]. In canine mammary tumors, the histological subtype has prognostic significance [31]. Similarly, in breast cancer of women, certain histotypes, including the tubular histotype, have a more favorable prognosis than invasive ductal carcinomas of no special type [32]. In rabbits the prognostic significance still has to be investigated.

Rabbit mammary carcinomas show a wide range of mitotic activity ranging from 0–36 mitotic figures in 10 high-power fields [10,11,26].

In two retrospective studies [11,26], the histological grade of rabbit mammary carcinomas is determined according to the grading scheme of Elston and Ellis [33]. This is an internationally accepted grading scheme for human breast cancer [32,34]. In breast cancer of women, the histological grade is an independent prognostic factor [34,35]. The prognostic significance of the histological grade in rabbit mammary carcinomas still has to be determined.

**Table 1:** Non-neoplastic proliferative and neoplastic mammary gland lesions in pet rabbits.

Mammary Lesion	Reference	Reported Frequency
*Non-neoplastic proliferative lesions*		
Lobular hyperplasia	[11]	2 of 124 rabbits
	[24]	1 rabbit (case report)
	[26]	2 of 24 rabbits
	[36]	17 rabbits (case series)
Multiple cysts	[11]	25 of 124 rabbits
	[26]	10 of 24 rabbits
Dysplasia	[24]	1 rabbit (case report)
	[37]	9 rabbits (case series)
Fibroadenomatous hyperplasia	[38]	20 rabbits (case series)
*Benign tumors*		
Tubular adenoma	[10]	3 of 109 rabbits
	[11]	2 of 124 rabbits
Cystadenoma	[11]	1 of 124 rabbits
	[26]	3 of 24 rabbits with 2-3 adenomas
Complex adenoma	[10]	1 of 109 rabbits
Intraductal papilloma *	[10]	8 of 109 rabbits
	[26]	1 of 24 rabbits with 2 papillomas
*Malignant tumors*		
In situ carcinoma	[11]	2 of 124 rabbits
	[26]	1 of 24 rabbits
Adenocarcinoma (different histotypes)	[10]	83 of 109 rabbits
	[11]	119 of 124 rabbits
	[24]	1 rabbit (case report)
	[26]	13 of 24 rabbits, 1 with 2 tumors
Complex adenocarcinoma	[10]	5 of 109 rabbits
Adenosquamous carcinoma	[10]	9 of 109 rabbits
	[26]	2 of 24 rabbits
Matrix-producing carcinoma	[26]	1 of 24 rabbits
Anaplastic carcinoma	[10]	3 of 109 rabbits
Spindle-cell carcinoma	[10]	1 of 109 rabbits
Ductal carcinoma	[10]	4 of 109 rabbits
Malignant myoepithelioma	[23]	1 rabbit (case report)
Carcinosarcoma	[25]	1 rabbit (case report)

* Synonymous designation: (intra)ductal papillary adenoma.

Rabbit mammary tumors and tumor-like lesions show the following frequent findings: Multiple proliferative non-neoplastic and neoplastic mammary gland lesions can occur together [11,21,23,26]. In particular, 20% of examined cases show the concurrent presence of mammary tumors and non-neoplastic cystic changes [11,26] and confirm the observations of Greene [21] (Figure 1). Therefore, a progression of non-neoplastic and benign lesions to carcinoma cannot be ruled out.

Non-neoplastic and neoplastic proliferative mammary gland lesions and the adjacent mammary tissue frequently display secretory activity characterized by the presence of proteinaceous material [11,21,26]. This finding is detected in the normal mammary gland parenchyma in 65%–90% of the cases, in 70%–100% of cystic lesions [11,26] and in 65%–100% of the mammary carcinomas [11,26] (Figure 1). This also confirms the findings of Greene [21].

**Figure 2:**
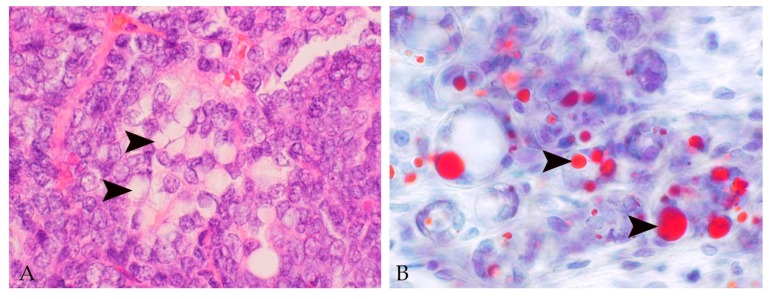
Microscopic images of rabbit mammary gland adenocarcinoma. (**A**) Adenocarcinoma with moderate numbers of tumor cells that contain well-demarcated empty vacuoles (arrowheads) (hematoxylin–eosin stain). (**B**) The Sudan-red stain on a cryostat section confirms that the vacuoles in tumor cells are lipid droplets (arrowheads).

Luminal epithelial cells of normal and hyperplastic mammary gland tissue and cystic lesions as well as epithelial tumor cells often contain cytoplasmic lipid droplets [10,11,26] (Figure 1 and Figure 2). Their presence is likely also related to lactational activity, since the rabbit milk has a high fat content [1,20].

The vast majority of rabbit mammary tumors are carcinomas [10,11,26]. In the referenced three retrospective studies, carcinomas encompass 50%–98% of the examined tumors, and adenocarcinomas predominate [10,11,26] (Figure 1 and Figure 2).

Tumors often show marked heterogeneity. For example, tissue invasion may be observed in some, but not all, cystic carcinoma areas (own observations). In addition, 89%–96% of carcinomas display more than one growth patterns [11,26]. Thus, in the case a larger tumor mass is submitted for microscopic evaluation, it is recommended to examine several sections of the same tumor for a concise histological assessment.

#### 4.2.2. Molecular Features

So far, very few investigations examined the molecular features of normal, proliferative and neoplastic mammary tissue in rabbits.

It has been shown that—similar to human beings [17,39], dogs [40,41] and cats [41,42]—myoepithelial cells in rabbits express the pancytokeratin marker AE1/AE3, vimentin, p63, SMA and calponin [26,43].

Immunostaining for myoepithelial markers assist the diagnosis of mammary non-neoplastic proliferative lesions, benign tumors, non-invasive cancer (in situ carcinoma) and infiltrative tumors not only in women [34,39,44], but also in dogs [40] and pet rabbits [26]. Whereas non-neoplastic lesions, benign tumors and in situ carcinomas of the rabbit mammary gland are completely rimmed by a single layer of non-neoplastic ME-cells, this layer is interrupted or absent in (invasive) carcinomas [26].

By hormone receptor binding assays, cytosolic receptors for estrogen and progesterone are detected in the mammary tissue of virgin, pregnant and lactating does [45]. During pregnancy estrogen and progesterone receptors decline, whereas during lactation estrogen receptors remain low and progesterone receptors are unmeasurable [45]. Bacci et al. [46] report the immunohistochemical detection of the estrogen-α receptor (ER-α) in two of three rabbit hyperplastic mammary glands, two of two adenomas and two of 10 adenocarcinomas.

In a larger retrospective study, immunostaining for ER-α and progesterone receptors (PR) confirms the expression of one or both receptors in normal mammary gland tissue (75%; 92 of 123 cases) as well as in mammary cysts (68%; 17 of 25 cases), lobular hyperplasia (2 of 2 cases), adenoma (3 of 3 cases) and in situ carcinoma (2 of 2 cases) [11]. In contrast, 63% of the invasive carcinomas are immunonegative for both receptors. In the latter study, no cut-off level is applied [11]. There is no statistically significant association between the ER-α and/or PR expression and the histological grade. A statistically significant correlation, however, exits between the reduced expression of ER-α and PR and an increased mitotic rate [11].

To the authors’ knowledge, human epidermal growth factor receptor type 2 (HER2) immunostaining on rabbit mammary tissue appears to have been only performed on a small number of cases [46]. One of two adenomas and one of 10 adenocarcinomas display a positive immunostaining [46].

#### 4.2.3. Evidence for a Hormonal Influence?

To our knowledge, no study has examined if mammary tumors in rabbits occur with equal frequency in female or female spayed rabbits. In bitches, early ovariectomy markedly reduces the risk of mammary tumor development [28].

The lack of expression of ER-α and/or PR in the majority of rabbit mammary carcinomas does not completely rule out an influence of estrogen and progesterone on tumorigenesis, since at least one of these receptors is expressed in 68% of the mammary cysts (*n* = 17) and all examined cases of lobular hyperplasia (*n* = 2), adenoma (*n* = 3) and in situ carcinoma (*n* = 2) [11]. The statistically significant increased mitotic activity in ER-α- and PR-negative tumors compared to those with expression of both receptors [11] argues against an influence of estrogen and progesterone on cancer progression.

The frequent lactational activity of rabbit mammary gland tissue with and without proliferative lesions [10,11,21,26] suggests a possible influence of prolactin on the development of mammary gland tumors and tumor-like lesions. This hypothesis is further supported by the following literature data:

Nine female nulliparous laboratory New Zealand white rabbits (2–4.75 years) with a pituitary gland adenoma of prolactin-secreting cells (prolactinoma) and markedly elevated serum prolactin developed non-neoplastic cystic changes in the mammary gland parenchyma associated with intraluminal papillary epithelial proliferations and lactational activity; the mammary gland lesions were diagnosed as dysplasia [37].

A 44-months old female laboratory New Zealand white rabbit with a prolactinoma and elevated serum prolactin was diagnosed with mammary hyperplasia, dysplasia and cystic mammary adenocarcinoma associated with secretory activity [24].

The application of the immunosuppressive drug cyclosporine to female nulliparous New Zealand white rabbits (daily dose: 10 mg/kg body weight, over 14 days) resulted in mammary gland hyperplasia and lactational activity [36]. Mammary changes were even more pronounced in rabbits with concurrent methylprednisolone application (daily dose: 5 mg per kg body weight) during the first two days of cyclosporine treatment [36]. The mammary gland changes were interpreted to be caused by the cyclosporine treatment, since the cyclosporine application was associated with increased serum prolactin, and the termination of the treatment resulted in a decrease in serum prolactin and a complete regression of the mammary lesions within 14 days [36].

Further, the application of cyclosporine to twenty male and female New Zealand White rabbits resulted in reversible non-neoplastic mammary gland changes diagnosed as fibroadenomatous hyperplasia in both genders [38].

Thus, it may be speculated that elevated prolactin levels stimulate non-neoplastic epithelial proliferation in the mammary tissue of rabbits leading to hyperplasia and dysplasia with a potential for neoplastic transformation in rare cases. In pet rabbits pseudopregnancy is a frequent condition that is associated with lactational activity and hyperplasia of mammary tissue [1,16,47]. Moreover, the onset of lactation is associated with a marked increase in prolactin receptors on rabbit mammary epithelial cells [48]. In the rabbit mammary gland at least three prolactin-binding subunits are detected [49]. Thus, additional studies are necessary to find out if does with frequent pregnancies or pseudopregnancies have a higher risk of developing proliferative mammary gland lesions.

Notably, in humans, prolactin is not only produced in lactotroph cells of the anterior lobe of the pituitary gland, but also in multiple other tissues, including the mammary gland [50,51,52] and uterus [51,52,53]. In the mammary gland tissue, which is also equipped with prolactin receptors, prolactin has not only endocrine functions, but can also evoke paracrine and autocrine effects [51,52]. In breast cancer of women, prolactin seems to show different actions, which are partially dependent on the remaining molecular status of the cancer type [54]. There is strong evidence that increased levels of prolactin or its receptor can promote cancer formation [51,52,54]. In established carcinomas, however, prolactin may facilitate tumor differentiation and thus it may reduce metastatic risk [55].

## 5. Discussion

### 5.1. Prognostic Biomarker

So far, histological and molecular features that predict the outcome of mammary tumors in pet rabbits are not available. The higher mitotic activity in ER-α- and PR-negative tumors in comparison to ER-α- and PR-positive mammary cancer [11] suggests a possible prognostic value of the mitotic count and the ER-α and PR status. The availability of a prognostic marker would be helpful for pet owners to make a treatment decision under consideration of animal welfare. This would be particularly important in aged rabbits or in those with other debilitating diseases. For some molecular markers cut-off values are established to predict treatment responses in women, e.g., a 1% threshold for immunohistochemical ER expression [56]. For rabbit mammary carcinomas, however, such cut-off values are not established. Similarly, endocrine therapy of ER- and PR-positive canine mammary carcinomas is not established in dogs [19]. Thus, it is recommended to report the results of the immunostaining for ER-α as percentages of mild, moderate and marked positive cells [19].

In triple-negative breast cancer of women (negative for ER, PR and HER2), the detection of certain components of the prolactin signaling pathway has likely prognostic significance [55].

### 5.2. The Rabbit as Animal Model for Breast Cancer in Women?

Rabbits are widely used in translational research for the reasons detailed below that also support their value for breast cancer research. They are phylogenetically more related to primates than rodents [6] and show similarities to humans regarding their immune system gene expressions, physiological functions and pathomorphological findings [5,6,57]. Housing and breeding are facilitated by their tame behavior, short gestation periods and average litter sizes of 4–12 pubs [5,6,8]. The size of rabbits permits sampling of blood, tissue or cells in sufficient amounts for most investigations as well as monitoring of therapeutic procedures [5,6]. The latter is also supported by the relatively long lifespan of rabbits [12] and the availability of comprehensive data on the rabbit genome and transcriptome [3,4,6,58]. Moreover, a common nutrition-associated disease in adult pet rabbits is obesity [8], that is a frequent life-style associated disorder in humans as well [59].

In addition, the histological structure of rabbit and human mammary gland complexes is similar, whereas the murine mammary gland shows marked morphological differences [57]. Thus, the rabbit has been suggested as a suitable animal model for the development of intraductal therapeutics to treat breast cancer [57].

In breast cancer of women, different molecular subtypes are distinguished based on the immunohistochemical detection of ER, PR, HER2, basal cytokeratins and the Ki-67 proliferation index [60]. A meeting abstract describes immunostaining of 10 rabbit adenocarcinomas for ER-α, the basal cytokeratins 5 and 6, as well as HER2 and the subsequent identification of molecular subtypes comparable to those in breast cancer of women, i.e., normal-like, luminal-like A, basal-like and HER2 overexpression [46].

For studies into prognostic markers on rabbit mammary carcinomas, important prerequisites would be a consensus histopathological classification, a standardized immunohistochemical procedure and evaluation scheme, as well as the set-up of long-term follow-up studies. In regard to the latter, the following aspects have to be considered:

Rabbits may show the concurrent presence of non-neoplastic, benign and malignant mammary lesions, and may be presented at different stages of the disease. Animals may be lost for follow-up because of their death due to other reasons than the mammary mass or because of relocation of the owners. The permission for a postmortem examination may not be obtained. Necropsy, however, would reveal with certainty the presence of additional mammary lesions, metastases or possible additional findings. Rabbits may show the concurrent presence of mammary cancer and uterine carcinoma. In case of lung metastases, it may not be possible to determine their tissue of origin, i.e., mammary carcinoma or uterine carcinoma.

### 5.3. Treatment

So far, the only treatment option for mammary masses in pet rabbits is surgical excision, that is performed under general anesthesia. The latter, however, may not be tolerated by debilitated rabbits with diseases of other organ systems, in particular, lungs, heart and kidneys. Thus, there is a need for additional treatment options in rabbits. It has to be considered, however, that despite similar receptor expression profiles in rabbit and human mammary tissue, treatment responses may differ. In women, ER-positive breast cancer is treated using substances with antiestrogenic functions, such as tamoxifen or aromatase inhibitors [34]. In intact bitches, tamoxifen treatment is associated with a high risk of pyometra [61].

To the best of our knowledge, the effect of tamoxifen on rabbit mammary tissue has not been tested so far. In rabbit uterine tissue, tamoxifen seems to exert antiestrogenic effects. It increased nuclear estradiol and progesterone receptors, whereas their cytosolic concentrations decreased [62]. Moreover, it stimulated the conversion of estradiol to a less potent estrogen derivate [62,63].

Therapeutic intervention of the prolactin receptor pathway will likely offer novel treatment options, in particular in triple-negative breast cancer of women [54,55].

## 6. Conclusions

This review summarizes the current scientific knowledge on mammary tumors and tumor-like lesions in pet rabbits and outlines practical aspects of the available information for treatment and wellbeing of rabbits. In addition, it highlights aspects of comparative pathology, particularly regarding breast cancer in women. Further research is needed; however, it will only be beneficial if the assessment of non-neoplastic and neoplastic proliferative mammary lesions in pet rabbits follows a consensus classification. In addition, to reveal if the rabbit can serve as an animal model for certain breast cancer types of women, molecular markers have to be established on rabbit tissue and evaluated by a standardized procedure.
